# Ultrafast Spectroscopy in Chemistry

**DOI:** 10.1021/acs.jpclett.6c01667

**Published:** 2026-07-28

**Authors:** Galina Grechishnikova, Gregory D. Scholes

**Affiliations:** † Department of Chemistry, 6740 Princeton University, Princeton, New Jersey 08544 United States

## Abstract

Ultrafast spectroscopy has become an indispensable investigation tool in numerous areas of chemical research encompassing purely synthetic to semiconductor material domains. Harnessing light on its femtosecond time scales offers a glimpse into submolecular processes that direct reaction pathways, energy transfer, charge separation, and many other fundamental chemical phenomena. In this Perspective, we highlight select examples that introduce how ultrafast spectroscopy became a pillar for modern chemical science. We emphasize the achievements of conventional pump–probe spectroscopy by showing its contribution to disentangling peculiar mechanisms of carrier relaxation in semiconductor nanocrystals, charge separation in organic photovoltaic devices, and photoredox catalyst activation. Our examples show that even traditional pump–probe experiments can provide extensive insights that go beyond the resolution of ultrafast time scales if combined with a thorough preliminary assessment of the target system. We conclude with suggestions for how ultrafast spectroscopy in tandem with chemical science can embark on advancing practical quantum information research.

## Graphical abstract

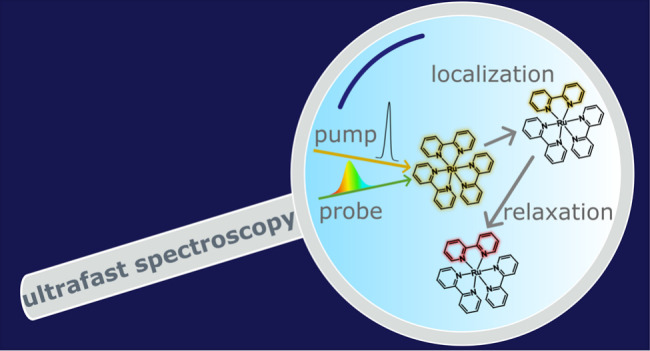

In the 21st century, chemical research became inseparable from the ultrafast investigation of the underlying atomic-scale processes with even synthetic fields widely embracing the method despite its complexity.^1–10^ Comprehensive investigation of nanoscale chemical processes often entails a coupled optical ultrafast study with proportional effort and time investment. What makes ultrafast spectroscopy such a powerful method for investigating transformations of chemical systems? How and why, despite being instrumentally and theoretically nuanced, has it become a conventional tool for the modern chemist? Can ultrafast spectroscopy go beyond resolving ultrashort time scales? This Perspective attempts to address these questions by highlighting a select number of studies that showcase the tightly coupled historical and modern development of optical methods and chemical science. We confine our Perspective to the visible, ultraviolet, and infrared (IR) regimes; however, frontier ultrafast investigations are not limited to these electromagnetic spectral ranges.

Accessing time scales shorter than those perceived by the eye or photography was achieved gradually.^11,12^ Instrumental and method development was driven by the need to understand increasingly shrinking fundamental chemical and physical phenomena. The development of lasers was a key advancement that enabled precise temporal and phase control of light, converting it to a coherent narrow beam of radiation. A modern ultrafast spectroscopy setup is inconceivable without a laser as an illumination source. However, even before this groundbreaking discovery in 1960,^13^ stroboscopic methods and flash photolysis by the means of flash lamps were performed to study chemical reactions.^14,15^ The emergence of laser technology resulted in a step function growth of ultrafast science, as eventually it provided precise control over pulse duration and intensity through mode-locking and pulse compression among other techniques.

The word “laser” originally started as an acronym for Light Amplification by Stimulated Emission of Radiation. Efficient generation of coherent photons occurs in materials, or gain media, that can sustain population inversion relative to the equilibrium distribution dictated by the Boltzmann law.^16^ Shifting this balance requires a particular energy level structure and is most effectively achieved in a four-level system (Figure 1A). While continuously pumped by light, often another laser, average populations are redistributed toward the longer-lived, or metastable, second excited state (Ex2) while the lowest excited state (Ex1) loses population quicker due to its fleeting nature. The prevailing process is determined by the difference between stimulated absorption and emission rates between these levels and depends on their occupation. Amplification of stimulated emission is achieved by placing the gain media in the resonator between two mirrors so that the light passes multiple times through the gain medium (Figure 1B).^17^ Passive mode-locking, commonly using a saturable absorber, allows for selectively extracting pulses with high peak intensity. Their duration is controlled by means of compression optics, such as chirped mirrors, grating, prism pairs, or fibers.^18^ Compression of laser pulses to femtosecond time scales and achieving the so-called transform-limited pulse duration led to profound advances for fundamental chemical research as it provided the ability to study processes that involve atomic nuclear motion and lie at the heart of chemical transformations.^2,19^ Molecular vibration frequencies are dictated by the atomic masses and bond force constants of the species involved and span the 10–100 fs ranges, making ultrafast femtosecond spectroscopy a perfectly suited direct tool to measure not just the chemical kinetics but also the dynamics of atomic and molecular scale processes.^20,21^

**Figure 1:**
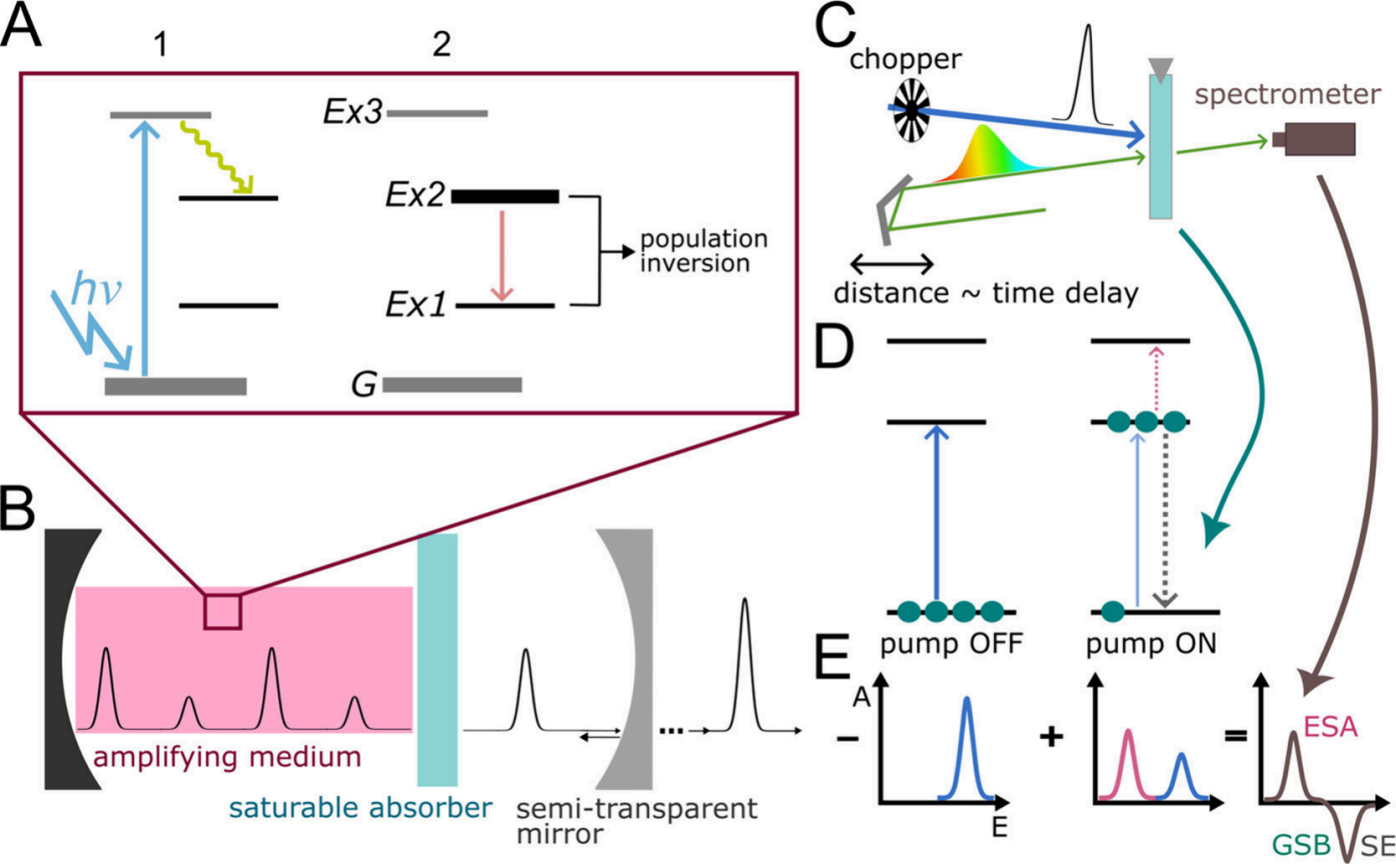
Spectroscopy fundamentals. (A) Population inversion in a four-level system. Laser excitation is denoted by *hv*. Scheme 1: Excitation to the higher excited state (Ex3) and subsequent relaxation to the long-lived second excited state (Ex2). The yellow arrow in scheme 1 corresponds to the nonradiative relaxation. Scheme 2: Stimulated emission from Ex2 leads to generation of coherent photons. The thickness of the energy levels reflects their relative population. G = ground state, Ex1 = short-lived lowest excited state. (B) Laser optical cavity. The initial pulse is enhanced in the amplifying medium. Low-intensity pulses are blocked by the saturable absorber. (C) Components of the ultrafast transient absorption setup. Pump (blue) and probe (green) laser pulses arrive at the sample with a set time difference. The time delay is controlled by the difference in traveling distance of the two pulses set by the retroreflector mirror on the mechanical stage. The transmitted probe is collected by the spectrometer. (D) Pump excitation brings the systems to the electronic excited state. Later, the probe pulse interacts with the system either further exciting it to the higher energy levels or stimulating emission. (E) Recorded pump–probe spectrum is the difference between absorption signal with pump ON vs pump OFF set by the chopper. Decrease in the ground state absorption caused by the population decrease appears as a ground state bleach (GSB) signal. It might overlap with stimulated emission (SE) from the lowest excited state. Excitation to higher excited states produces excited state absorption (ESA) feature.

Today, the most widely used ultrafast nonlinear spectroscopic method is transient absorption spectroscopy, a kind of pump–probe experiment. It combines the advantages of a nonlinear technique with the potential for physically transparent data interpretation that does not require a higher-order spectroscopy formalism.^1,4^ TA spectroscopy involves two laser beams, commonly referred to as the pump and probe beams, that respectively excite the system and monitor its excited state after a known waiting time termed the pump–probe delay time (Figure 1C). Upon photoexcitation by the pump, the system evolves during the pump–probe delay time, redistributing population from the initially excited state by following the available transfer pathways, i.e., transitions to the lower lying excited states or back to the ground state. After the waiting time passes, a probe pulse interrogates the system, capturing a snapshot of chemical evolution at a certain time. While the delay between the pulses is varied, a collection of such snapshots allows one to recreate and map various excitation and relaxation routes that the system follows to reach equilibrium. A common way to report transient absorption spectroscopy results is to separate them in spectral and temporal domains, with the former plot reporting on the species that emerged during the measurement and the latter showing their time evolution recorded at select wavelengths. Coupled together, such data representation points to the nature of the inherent states of the system.

Expanding ultrafast spectroscopy beyond the pump–probe regime was catalyzed by a multitude of unresolved questions—mainly regarding fundamental mechanisms of photosynthesis.^22^ At the same time, since the early stages of photosynthetic research, pump–probe spectroscopy was essential for experimentally verifying predictions of long-range energy transfer in pigment–protein complexes made decades before the first X-ray structure of such a system had become available.^23,24^ In the 1980s, emerging spectroscopic data and X-ray crystal structure of photosynthetic units suggested highly efficient energy transfer between densely packed molecules held together by the protein environment. Solutions of equally concentrated molecular chromophores displayed rapid quenching of excitation upon illumination.^25^ Thus, the role of the protein scaffold and its interplay with pigments in the photosynthetic pigment–protein complexes became one of the central questions of ultrafast chemical science. The abundance and proximity of chromophores in pigment–protein complexes induced multiple overlapping pump–probe signals from similar but not identical, i.e., disordered, pigment units and resulted in congested spectra. Resolving contributions from individual chromophores was accomplished by introducing another dimension to the pump–probe spectroscopy: excitation frequency.^26,27^ By correlating excitation and emission energies of the chromophores and recording their evolution in time, it became feasible to track excitation energy pathways between molecules in a pigment–protein complex. This solution, although elegant, came at the cost of instrumental complication requiring cumbersome alignment and additional phase stabilization of laser pulses. Advancement of this two-dimensional approach further resolved distribution of chromophores in pigment–protein complexes by correlating vibrational motion with electronic structure of the molecule.^28,29^

Open questions regarding mechanisms of energy transfer in pigment–protein complexes facilitated the coevolution of ultrafast spectroscopy and molecular dynamics.^22^ Counterintuitively, photosynthetic systems ultimately proved to be perfect models of interchromophore interactions owing to the external geometric constraints imposed by the protein environment. Energy transfer in tightly arranged structures could not be modeled using conventional dipole–dipole interactions as often distances between the molecules and their sizes were on the same scale.^30^ Instead, more sophisticated approaches emerged that took into account uneven electron density distribution within the interacting molecules, leading to resolving highly congested energy transfer pathways in photosynthetic systems.

A pivotal advancement of nonlinear spectroscopies that enabled predictive capability and introduced a common language for all ultrafast methods was the development of theoretical foundations for nonlinear interactions of light fields with matter.^31^ In the 1990s, the further improvement and expansion across disciplines of laser technologies led to experimental works utilizing various configurations of multiple laser pulses and thus created signals with an ambiguous interpretation. Compounding the problem, such results were hard to compare across different published works. To address this issue, Mukamel formulated a generalized framework of nonlinear spectroscopies in 1995. The formalism, although underutilized outside of the field of ultrafast spectroscopy, offered a clear connection between experimental techniques and the physical insights they could provide. Nonlinear methods were categorized by a characteristic number of pulses and their time ordering when interacting with the sample. For example, in this framework, TA spectroscopy was regarded as a third-order nonlinear spectroscopy that involves four light–matter interactions (first two from the same pump beam) and two delay times (with the first time delay between identical pumps effectively zero) (Figure 1C). The response of the system under study was introduced through the induced polarization, and its time evolution under the radiation field was treated perturbatively. Such a sophisticated approach to the body of nonlinear techniques provided not only explanatory but also predictive power for the works to come.

While ultrafast spectroscopy impacted a vast majority of chemical research directions, providing nuanced details on atomic-scale phenomena, in the following Perspective we focus on the exemplary cases that demonstrate both the capabilities and limitations of the ultrafast methods in a highly concentrated manner. We first delve into the advancement of quantum dot carrier dynamics, as these studies serve as a unique example of the layered development of the now-mature field catalyzed by insights from ultrafast optical investigations. Next, we discuss surprisingly fast and efficient charge separation in organic photovoltaic materials. Then, we explore the rapidly developing field of photoredox catalysis. Finally, we touch upon the current challenges of the ultrafast spectroscopy discipline. We highlight select works with contributions to each of the above areas of chemical science. We focus mainly on achievements of transient absorption spectroscopy since its relative accessibility and versatility make it the most widely used nonlinear ultrafast technique today.

A particularly prominent example of how ultrafast spectroscopy has spurred the development and application of chemical science is the advancement of nanoparticle research.^32–36^ Pump–probe spectroscopy proved to be an invaluable tool not only for discerning fundamental mechanisms that govern carrier generation and relaxation in nanostructures but also for probing and quantifying integral application-relevant parameters. The profound impact of the discovery of quantum dots (QDs), or semiconductor nanocrystals, merely half a century ago closely resembles that of laser development in 1960. While laser technologies allowed for harnessing light on ultrafast time scales, giving access to the submolecular femtosecond regime, QDs enabled precise control over excited quasiparticles on their intrinsic spatial scales, vaulting toward experimentally observable quantum mechanical effects. Nanometer-size structures have artificial atom-like properties and operate in a regime where electron and hole Coulomb interactions and confinement energies have comparable magnitudes due to wave function localization, which is determined by the QD size.^37–40^ While a simple particle-in-a-box model can qualitatively explain quantum confinement effects in QDs, such as discretization of energy levels and size-dependent optical properties, it fails to accurately predict physics that emerges due to particle interactions. More sophisticated theoretical approaches, some relying on the effective mass approximation, are required to capture Coulomb interactions, asymmetric band structure, wave function leakage, surface effects, and crystal symmetry in QDs. The fundamental importance of many of these phenomena was first realized through optical studies of QDs that revealed surprising correlations or lack of correlations between the physical structure of a QD and its intrinsic properties. Moreover, thorough theoretical studies in conjunction with ultrafast investigations allowed one to quantitatively interpret observed spectroscopic results and uncover hidden nuances of QD phenomena, taking pump–probe investigations beyond discerning the dynamics of excited species.

Initially, producing samples with narrow size distribution of QDs created a bottleneck for abundant experimental investigations of nanostructures, but theoretical works were not constrained by the absence of high-quality specimen and thus set the groundwork for the ultrafast studies to come. One of the first discrepancies between theoretical predictions and experimentally observed outcomes addressed by ultrafast spectroscopy was carrier relaxation in QDs. Single excitons, i.e., bound excited electron and hole pairs well-separated from other excited species, are generated under weak excitation conditions, a regime relevant for solar cell applications of QDs. It was anticipated that quantization of energy levels within the conduction and valence bands of the QD and ensuing energy and momentum restrictions, a consequence of quantum confinement, would result in the “phonon bottleneck” effect present during carrier relaxation (Figure 2A). In bulk semiconductors excited electrons with excessive kinetic energy relax to the conduction band edge through interactions with quantized optical phonon modes. Substantial separation in energies in QD bands requires multiple phonon quanta to bridge this gap. Such a constraint was expected to inhibit this route of carrier relaxation leading to prolonged lifetimes of excited species. However, experimental ultrafast spectroscopy investigations detected carrier relaxation rates on the picosecond time scales, similar to that of bulk materials, contradicting the presence of the “phonon bottleneck” effect. Moreover, excited electron and hole seemed to follow distinct relaxation pathways.

**Figure 2:**
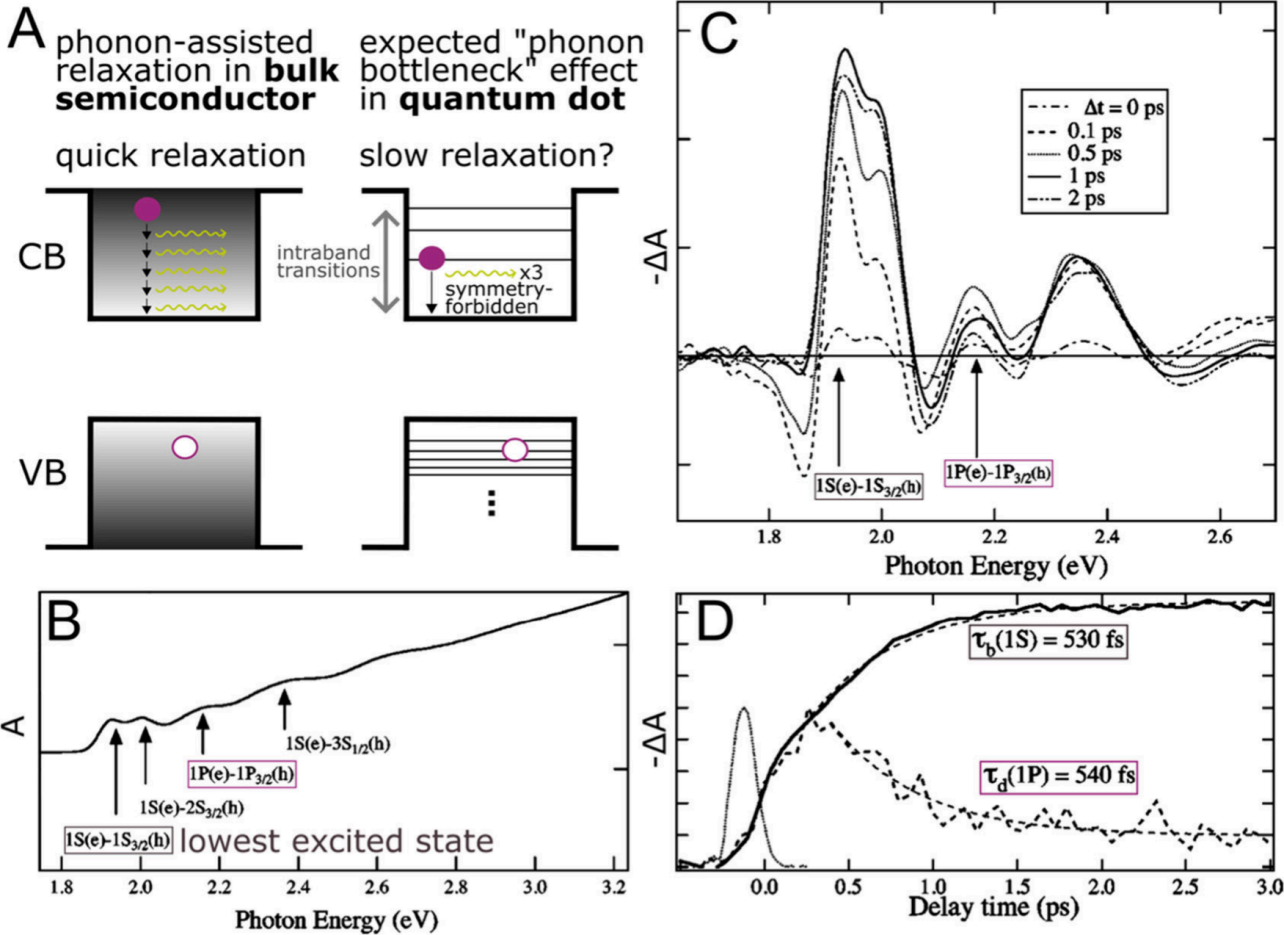
Single carrier relaxation in semiconductor quantum dots. (A) Discretization of energy levels in QDs was expected to result in the “phonon bottleneck effect”. VB = valence band, CB = conduction band. (B) Absorption spectrum of a CdSe QD showing multiple excitonic transitions. (C) TA spectra recorded at different early time delays. (D) TA dynamics showing subps decay of higher exciton and concurrent population of the lowest excitonic state. (B), (C), and (D) are adapted with permission from ref 41. Copyright 1999 by the American Physical Society.

Careful examination of CdSe QDs excitation via visible and near-IR TA spectroscopy allowed for the disentangling of electron and hole relaxation channels and the probing of the “phonon bottleneck” effect (Figure 2).^41^ Sensitivity of electron excitation to the state filling effect, i.e., constrained occupation of discrete energy levels due to the Pauli exclusion principle, provided the necessary selectivity for the assignment of visible TA bleaching features as mainly electron dominated. At the same time, although IR TA was sensitive to intraband transitions of both electrons and holes at each wavelength, a slow relaxation component correlated with electron dynamics was discerned from the visible TA experiment and thus the residual fast decay was attributed to the hole relaxation. Recognizing the importance of surface defects in QDs due to large surface-to-volume ratio, Bawendi et al. compared QDs with different surface passivating agents revealing that, in contrast to excited electron, hole relaxation pathways are unaffected by surface trap states and follow intrinsic relaxation routes.^41^ The absence of the phonon-mediated relaxation pathways suggested that other routes, potentially relying on Auger-type interactions, are responsible for picosecond (ps) carrier relaxation lifetimes in nanocrystals. Later ultrafast investigations of QD carrier relaxation dynamics provided a wealth of evidence that multiple excitation decay mechanisms could simultaneously be at play in these nanostructures. The nature of quantum dots and their structural properties, such as size and ligand passivation, determined the prevalence of a certain relaxation mechanism. Excessive electron energy could be dissipated through Auger interaction with a hole and subsequent phonon relaxation, via trap states, or vibrational modes. QD design geared toward minimizing ligand and environment interactions of the QD core by introducing a thick shell proved to be a feasible practical strategy to slow down carrier relaxation rates from picosecond to nanosecond ranges.^42,43^

Despite being equally if not more challenging, understanding ultrafast QD carrier relaxation dynamics in higher excitation density regimes yielded meaningful insights for the functional design of QDs essential for their realization as optical gain media.^10^ Restricting the semiconductor energy landscape in three dimensions generates discrete energy levels with high oscillator strengths; combined with high structural tunability, these properties make QDs seemingly perfect for this application. However, excitation of QDs with high-power light results in multiple excited species occupying rather limited nanostructure space, leading to strong Coulomb interactions between them. As a result, multiexciton relaxation dynamics is confined to the first hundreds of picoseconds with single exciton response prevailing at later times, making this beneficial effect impractical. Further, using a state-resolved ultrafast TA approach, P. Kambhampati and co-workers were able to separate the dynamics of two different biexciton species, showing that pure biexciton decays within the first picosecond after photoexcitation and attributing the leftover longer-time picosecond response to the mixed trapped surface-core biexciton.^44,45^ Still, identifying processes and structural characteristics that govern ultrafast multiexciton relaxation provided invaluable information for practical development of QDs. As the field advances and QD design becomes more intentional, QD structures arise that suppress fast multiexciton relaxation pathways, for example, by physically separating electron and hole, paving the way toward feasible optical gain in QDs.^46^

Another promising practical aspect of QD carrier dynamics predicted and discovered by the Los Alamos National Laboratory and the National Laboratory of the Rockies (formerly NREL) scientists was the carrier multiplication or multiple exciton generation phenomenon (Figure 3). This effect was suspected due to the confinement-induced strong Coulomb carrier interactions and aimed to tackle the problem of the intrinsic limit on the solar cell efficiency by converting one high-energy photon into multiple band-edge excitons. The simultaneous suppression of the phonon bottleneck effect and presence of strong Auger recombination channels pointed toward the possibility of hot electron relaxation through the transfer of the excessive energy to a secondary charge carrier.^47^ Such an interaction could excite another electron across the band gap and result in the increased solar cell photocurrents. The carrier multiplication effect was detected experimentally using ultrafast transient absorption spectroscopy with pump energies equal to the multiples of the band gap separation.^48,49^ The strategy was to track early time changes in the bleach signal of the band-edge exciton as the pump energy increased and then compare them to the on-resonance excitation corresponding to the single carrier generation regime. An increased bleach signal indicated that more than one excited carrier is produced per one absorbed photon. However, real multiple exciton generation has so far proven to be hard to implement in practice, mainly because multiple exciton states decay quickly. However, iterative elucidation of its nuances has emphasized and instilled principles of thorough and deliberate spectroscopic analysis.^50^ Variables such as excitation energy and power, stirring the colloidal solution, heterogeneous sample variability, and explicit knowledge of the system prior to the ultrafast TA work have been shown to be essential components for obtaining accurate quantitative results.

**Figure 3:**
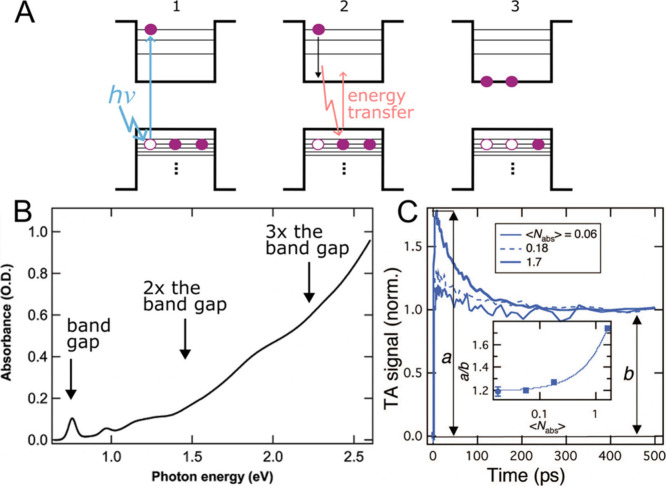
Quantification of carrier multiplication effect in quantum dots. (A) Surplus excitation energy (pink) is transferred to another electron via Coulomb-mediated interaction. (B) Pump energies reflecting multiples of band gap separation. Adapted with permission from ref 49. Copyright 2005 American Chemical Society. (C) TA dynamics showing fast biexciton decay to the single excitation state for different pump intensities. Inset: number of excited species per quantum dot (*a*/*b*) as a function of number of photons absorbed (⟨*N*_abs_⟩). *a* = early time transient absorption amplitude containing contributions from both multiple and singlet excitons; *b* = long-time signal reflects single exciton dynamics. Reprinted with permission from ref 48. Copyright 2008 American Chemical Society.

An additional factor that caused ambiguity in interpreting and comparing ultrafast spectroscopy results across different studies is the intrinsic fine structure of the excitonic states of QDs. Most directly evidenced by a Stokes shift between QD steady-state absorption and emission spectra, this subtle feature of QD energy structure influenced carrier relaxation dynamics and optical gain investigations, as established by the state-resolved TA approach carried out by P. Kambhampati and co-workers^51,52^ found that electron and hole exchange interactions mix their angular momenta and split the lowest excitonic state of CdSe QDs into multiple bright and dark states.^53^ These transitions cannot be observed selectively in conventional ultrafast transient absorption due to the congestion of the underlying spectral features. However, the spin-flip dynamics between these closely spaced states was discerned using ultrafast cross-polarized transient grating spectroscopy (Figure 4).^54^ This nonlinear technique involves three laser pulses: two pumps arrive at the sample simultaneously, creating a grating, and later, a probe pulse diffracts off the grating and tracks changes in the sample. The linear cross-polarized pump regime was shown to be sensitive to the spin-flip dynamics of bright QD fine structure states. Ultrafast transient grating allowed us to study colloidal solutions of randomly oriented QDs at room temperature since signal amplitude was sensitive to the change in the exciton spin during the time between generation of the grating and its subsequent interrogation by the probe and did not require the imbalance between the different spin states.^55^ Extensive data modeling revealed that all eight states participate in the spin exchange interactions with the dominant processes and their rates determined by the DQ size.^56^ QDs of all sizes exhibited fast subpicosecond kinetic component that was dictated by the hole relaxation and resembled intersystem crossing in molecules since this transition occurred between excitonic states with different total angular momentum. Larger QDs displayed an additional slower process with internal conversion-like character. These findings allowed us to relate the fast component spin flip rate to the effective confined exciton size in QDs.^57,58^ Cross-polarized transient grating is a unique example of optical ultrafast investigation of exciton fine structure spin dynamics with linearly polarized light.

**Figure 4:**
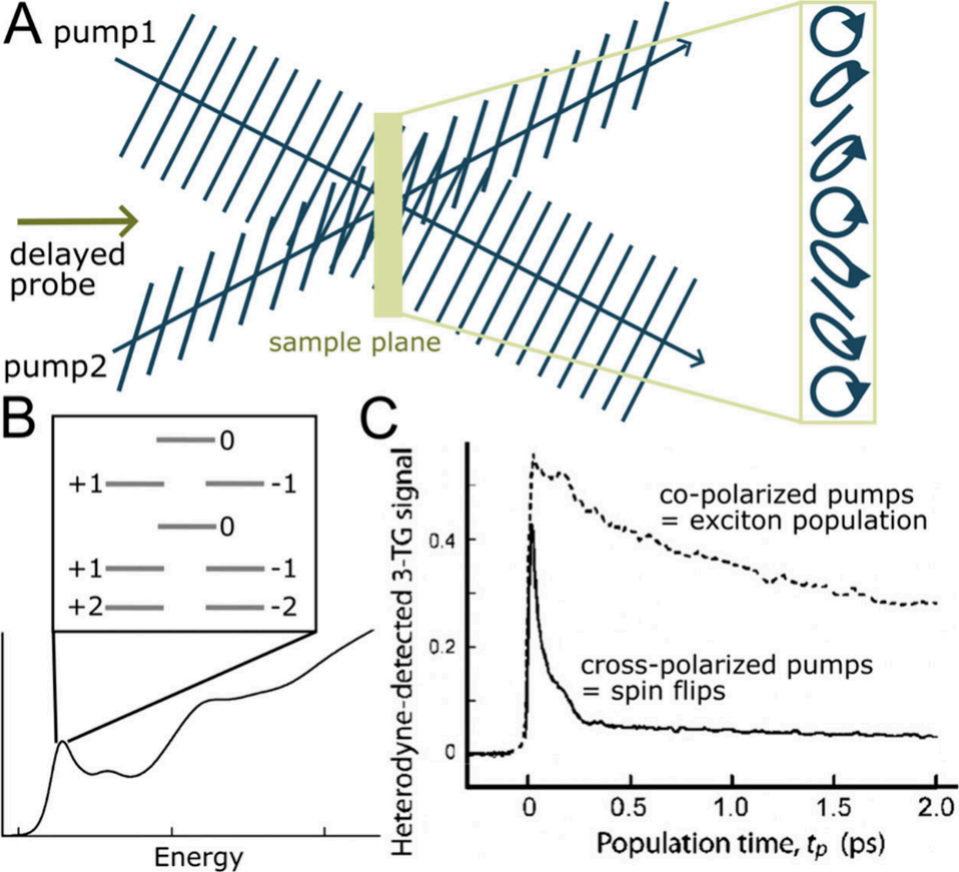
Spin-resolved dynamics of QD lowest exciton fine structure. (A) Schematic of the transient grating formed in the sample plane. Linearly cross-polarized (orthogonal) pump pulses create periodic changes in polarization. Later, probe diffracts off the dynamic grating. (B) Absorption spectrum of CdSe QD. Lowest excitonic state of QD is split due to electron–hole exchange correlations producing states with different total angular momenta. Adapted with permission from ref.^59^ Copyright 2006 American Chemical Society. (C) Transient grating signals obtained with co- and cross-polarized pump pulses in a heterodyne detection configuration. Adapted with permission from ref 54. Copyright 2006 by the American Physical Society.

As the QD field matures, correlations between the structure of the nanocrystal and its intrinsic properties deduced from ultrafast examinations become more apparent.^60^ While initial ultrafast studies of QDs focused on understanding fundamental mechanisms that govern carrier generation and relaxation, modern works iron out key nuances of these processes and address the gap between proof-of-concept studies and the ubiquitous practical implementation of QDs in solar cells, displays, fields of biological imaging, sensing, and quantum technologies.^61–68^ In combination with other experimental techniques, ultrafast spectroscopy provides information on carrier diffusion, role of energetic and structural disorder, and single-particle interactions expanding the QD application range.^69–71^

As QDs provide high structural and optical control compared to bulk semiconductors, organic photovoltaic (OPV) materials, similarly, were originally devised as a more tunable and cheaper alternative to the conventional silicon solar cell.^72,73^ In contrast to seamless charge separation in silicon, attributed to its high dielectric constant, in organic-based semiconductors photoexcited electrons and holes are strongly bound by Coulomb interaction making separation of charge carriers tricky. Initially, creative design strategies were required to circumvent this and other limitations of organic materials and facilitate their practical implementation. In particular, efficient charge separation in OPV systems was achieved by incorporating a dual-film architecture where electron and hole splitting was facilitated at the interface due to the energy level offset between organic compounds, namely, electron donors and acceptors. This elegant solution, however, required further optimization due to the short excited-state lifetimes limiting the maximum diffusion distance of carriers and thus thickness of the films to ∼10 nm, while ∼100 of nm was required for the OPV devices. A simple yet brilliant workaround was to blend two materials together, increasing the interface area between the electron donor and acceptor domains (Figure 5A). Moreover, the size of the domains was on the order of 10 nm, small enough for charges to reach the interface for further separation without detrimentally recombining inside the domain. Ultrafast spectroscopy again proved to be an invaluable probe of intricate charge separation in these materials, revealing insights into the early time carrier dynamics and efficiency.^74–82^

**Figure 5:**
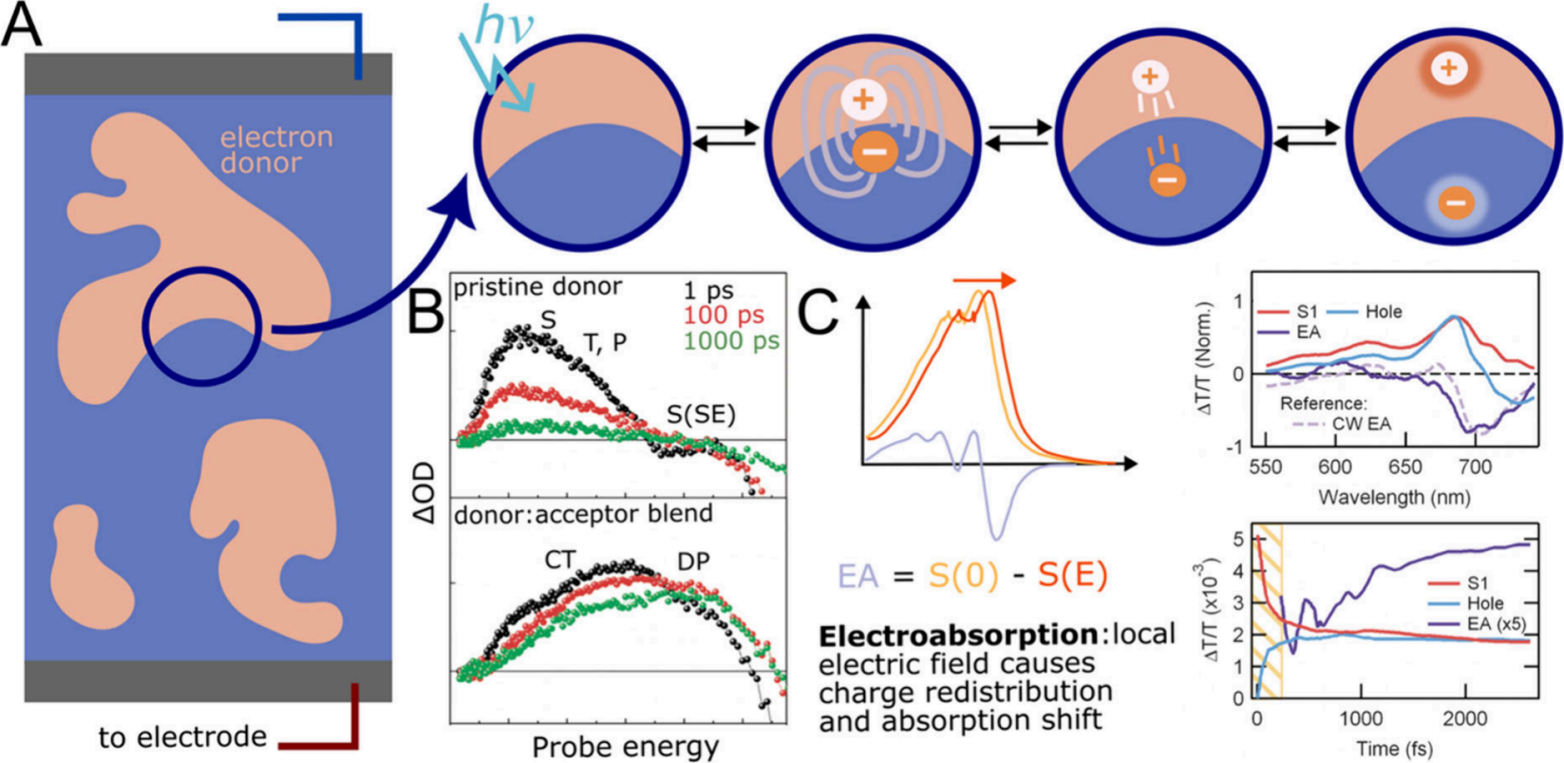
Charge separation in bulk heterojunction. (A) A blend of organic electron donor and acceptor materials in a bulk heterojunction device. A close-up view shows the principal ultrafast charge separation steps. Exciton migrates to the donor–acceptor interface upon laser illumination. Fast diffusion of the charged particles into separate domains precedes their stabilization by the polaron cloud. (B) Transient absorption spectra of pristine donor (top) and donor: acceptor blend (bottom) demonstrating formation of new long-lived charge separated (CT) state in the blend. S = singlet, T = triplet, P = polaron, SE = stimulated emission, DP = delocalized polaron. Adapted with permission from ref 83. Copyright 2014 American Chemical Society. (C) Sub-200 fs charge separation through the delocalized acceptor states tracked by electroabsorption signal. Origin of electroabsorption feature (left). Transient absorption spectra and dynamics of exciton (S1), hole, and electroabsorption (EA) components of global fit analysis. Modified with permission from ref 84. Copyright 2013 by the AAAS.

One of the early ultrafast transient absorption studies of a blended OPV system containing P3HT polymer as an electron donor and PCBM fullerene derivative as an electron acceptor aimed to elucidate the mechanisms of charge carrier generation and separation in the material.^83^ Key findings of this work are summarized in Figure 5B. Multiple bands are present in the TA traces of the control pristine P3HT film and P3HT:PCBM blend. The reference P3HT film signal decays within 1000 ps and contains contributions from singlet (S) and triplet (T) excitons and localized polarons (P2). In contrast, the blend TA signal persists beyond the experiment time window and displays novel spectral features assigned as a bound charge transfer (CT) and delocalized polaron (DP2) states. Additionally, TA traces of the annealed P3HT:PCBM blend exhibit identical transitions with more pronounced separation. Of particular significance is the time scale differences of the described experiments. The first major implication of this work is the profound difference between pristine and blend samples, even in the first 1 ps spectra, supporting earlier findings. This suggests that charge formation in the OPV material occurs on the subps time scale and electron and hole separation must follow similar ultrafast rates to be efficient. Next, charge-carrier separation evidently occurs in two steps. The bound charge transfer state that is formed upon photoexcitation decays within 9 ps in the blend film and 4 ps in its annealed version, forming delocalized polaron species that potentially transfer carriers to the separated domains. Reduction of the recombination loss by 2.5 times in the annealed blend is thus attributed to the shorter time window for the recombination of electron and hole while in the bound charge transfer state. This extensive study demonstrates the utility of ultrafast spectroscopy for discerning the ultrafast dynamics of charge generation and separation in blended OPV materials.

Later ultrafast spectroscopy work addressed subps processes that precede the formation of the bound charge transfer state (Figure 5C).^84^ Careful separation of time scales and global analysis of the TA data revealed three processes in the isolated dynamics of the excitons generated at the heterojunction. Two of them included generated singlet exciton and hole dynamics with 82 fs and 22 ps time constants. Interestingly, a third feature appeared exclusively in the sample containing fullerene acceptor domains and constituted an optical electro-absorption response of the system. Such an effect occurs due to the impact of the photoexcited charge carrier electric field on the nearby species, shifting their transition energies and resulting in the derivative-like features in TA spectrum. Moreover, by using electrostatic modeling it became possible to correlate this feature with the charge separation distance that increased from 1.5 to ∼5 nm when fullerene aggregates were present in the sample. Additional modeling provided a physical explanation for the observed early TA responses. It was shown that, in the first 200 fs, charge splitting happens due to electron interaction with the delocalized π-electron states of the fullerene acceptor, while at later times the polaron cloud catches up with charged carriers, localizing their states and charge transport at longer waiting times occurs through a hopping mechanism. This work provided a clear explanation of the long-standing controversy regarding extremely efficient charge separation in blended OPV materials despite disorder-induced localization and polaron formation processes—charge separation essentially occurs prior to these detrimental processes.

Eventually, despite the substantial progress, intrinsic limitations on the power conversion efficiency of fullerene-based OPV devices set at ∼13% stimulated the search for new OPV acceptor materials. This time, however, the screening was more specific as it was building upon a body of previous works on charge separation at the bulk heterojunction interface. Since 2015, non-fullerene acceptors, mainly molecules of the acceptor–donor–acceptor architecture that have high dielectric constants and wide light absorption ranges, are considered the gold standard for OPV devices, with their power conversion efficiencies reaching 20%.^85,86^ This new type of electron acceptors prompted another cycle of ultrafast charge separation investigations aiming to explain such remarkable efficiencies.^87^ Surprisingly, different mechanisms were at play in non-fullerene acceptor systems. While more evidence was accumulated that exciton separation in these structures is mediated by holes as opposed to electrons in fullerene-based OPV materials, it became apparent that efficient charge separation is taking place within the acceptor itself, and the presence of electron donor prevents detrimental recombination processes.^88,89^ This revised understanding of exciton separation in non-fullerene OPV materials, facilitated by ultrafast spectroscopy, opened the door for new device architectures including single-component and bilayer frameworks. As OPV materials settle into their niche of flexible and light solar energy converters, ultrafast spectroscopy in tandem with electrical and electrochemical methods continues to facilitate such an advancement.

A plenitude of chemical compounds with wide structural variation and abundance of inherent processes inspired further attempts to overcome intrinsic limitations of the artificial solar cell devices commonly referred to as the Shockley–Queisser limit.^90^ This theoretical value of ∼33% describes the efficiency of an ideal single bandgap solar cell. Since sunlight has broad spectral coverage, it is inevitable that material bandgap will not cover the whole spectrum with sub-bandgap photons passing through the material and above-bandgap photons absorbed but releasing excessive kinetic energy as heat losses. Electron and hole recombination can result in additional voltage decrease in the device. A process of singlet fission was proposed as a promising route to circumvent dissipation of extra photonic energy as heat for the above-bandgap excitation with a potential increase in maximum solar cell efficiency to 45%.^91,92^ As recognized today, this phenomenon includes a plexus of ultrafast interactions that result in high-energy singlet exciton dissociating into a pair of lower-energy triplet excitations.^93–100^ This conversion can effectively double the number of mobile charge carriers increasing photocurrent in the energy converting device.

As OPV devices progress toward practical implementation, more material aspects become essential to their efficient performance.^101^ Actual realization of singlet fission compounds in devices, such as solar cells, requires not only long generated carrier lifetimes but also their effective transport across the structure. Otherwise, slow diffusion of generated excited species can lead to the decreased efficiencies of the OPV system. Triplet configurations in singlet fission molecular materials, mostly dark states with small transition dipole moments, rely on the Dexter energy transfer mechanism which itself strongly depends on the wave function overlap between the states^102,103^ and is prone to disorder as compared to the hopping-like transition-dipole-moment-mediated Förster resonance energy transfer available to singlet carriers.^104–106^ Thus, it has been debated that an increase in total efficiency of a singlet-fission-based solar cell through utilization of triplet excitons might come at the cost of fast energy transport through the device.^107^ To resolve this ambiguity, coupling spectroscopic investigations with spatial imaging capabilities added an additional degree of control to material studies. Transient-absorption-based microscopy proved to be invaluable for singlet fission energy transport investigations as it could directly detect migration of optically dark triplet species (Figure 6).^108–112^

**Figure 6:**
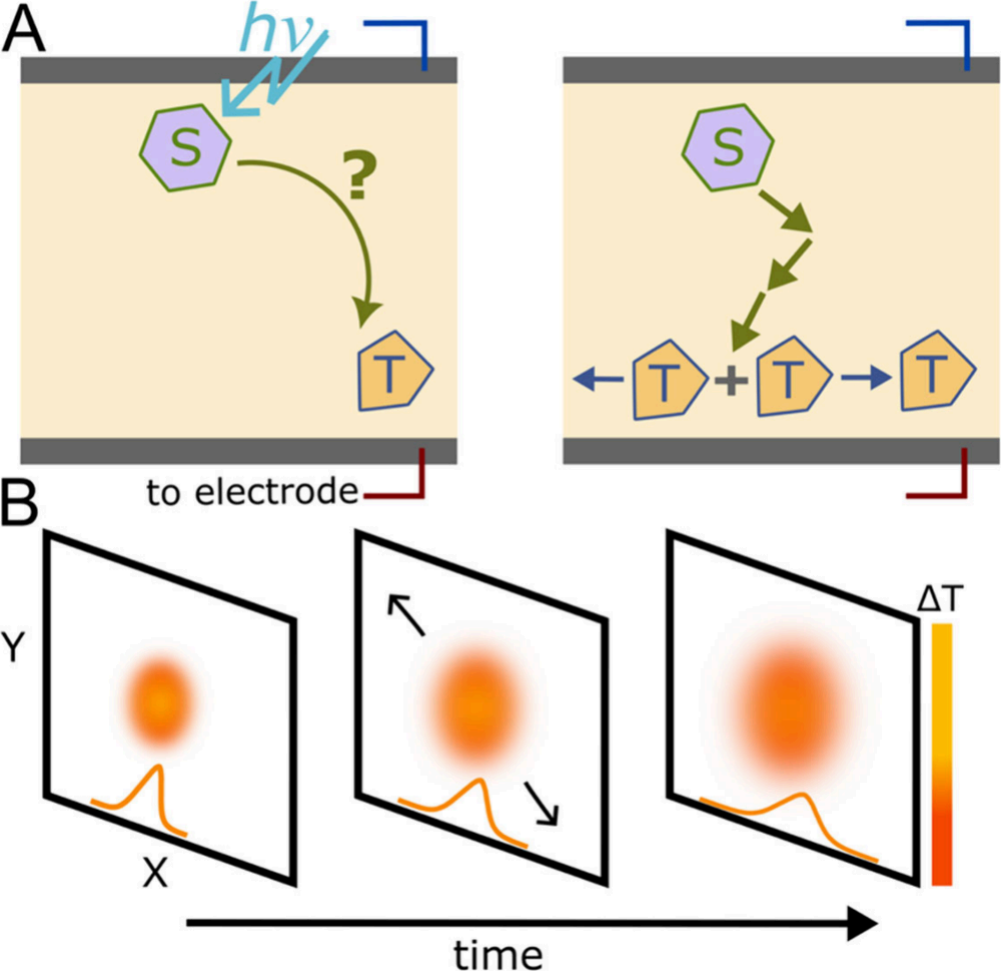
Singlet-mediated triplet exciton transport. (A) Dark triplet states generated via singlet fission diffuse to the electrode despite the delicate nature of the Dexter mechanism. (left) Exciton migration is mediated by singlets on short sub-100 ps time scales. (right) (B) Principle of transient absorption microscopy. Nonlinear spectroscopy provides contrast for tracking spatial evolution of excited species in time.

Contrary to the expectations that delicate mechanism of triplet energy transport would inhibit diffusion in the materials, initial time-resolved delayed fluorescence studies reported long-range overall diffusion of triplet carriers that was attributed to longer lifetimes of migrating species.^113,114^ To address this ambiguity, transient absorption microscopy (a combination of ultrafast transient absorption with spatial imaging) was used by L. Huang and co-workers studied triplet energy diffusion in common singlet fission materials, such as tetracene, rubrene, and TIPS-pentacene single crystals.^108,109^ By leveraging the almost unique capability of transient absorption to access ultrafast excited-state dynamics of dark triplet states, it was possible to separate singlet and triplet contributions to the energy transport in these compounds. Intriguingly, long-range triplet energy propagation developed as a cooperative result of intertwined singlet and triplet exciton diffusion with singlet-dominated transport at early (<100 ps) times. The moderate presence of a singlet population due to backward processes of triplet fusion and annihilation, typically seen as deleterious, enhanced triplet transport lengths. Monitoring singlet- and triplet-exciton migration at their corresponding excited-state absorption wavelengths provided direct evidence for singlet-mediated triplet transport. Moreover, comparison of materials with different singlet–triplet pair energy gaps suggested such an endothermicity difference as another engineering handle for singlet fission material design.

Proliferation of the ultrafast spectroscopic techniques in the 1980s also allowed us to examine chemical reactions in the liquid phase bringing to light the role of solvation and solvent reorganization in electron transfer reactions and transition-metal photochemistry.^115–117^ In the late 90s, thorough examinations of transition metal complexes of precious metals such as Ru and Ir that were central for many photochemical processes and later became essential for the field of photoredox catalysis laid the foundation for the rational design of photocatalysts. Photocatalysis, as opposed to thermally initiated chemical transformations, leverages light energy as a launching impulse for the reaction.^118,119^ While both thermal and light activation provide extra energy to the reaction, irradiation with light can selectively excite photocatalytic species, resulting in higher desirable product yields and milder reaction conditions. Following irradiation, the photocatalyst is promoted to its highly active electronically excited state, or it transforms into a radical and mediation of the photoredox reaction through electron or energy transfer becomes feasible. That enables processes such as bond homolysis and cross-coupling reactions, among others. As this dynamic discipline has been going through a revival since 2008, a growing number of intricate approaches to perform small-molecule transformations have emerged that require detailed mechanistic investigations on ultrafast time scales.^6,7,120–124^

While some state-of-the-art spectroscopic studies cover all steps of a photoredox process, including photocatalyst activation,^125–129^ the reaction itself,^130–132^ and unproductive pathways, early works focused specifically on the photoexcitation of the precious metal complexes and challenged the commonly accepted view that the outcomes of the photochemical process are entirely determined by the properties of the photocatalyst’s long-lived excited state. The unique capability of ultrafast spectroscopy to probe the dynamics of fleeting species on ultrashort fs–ps time scales was leveraged in one of the first fs transient absorption studies of the excited state of [Ru­(bpy)_3_]^2+^ that revealed multiple rearrangements of this complex upon photoexcitation (Figure 7).^133^ Interestingly, transition from the initially excited bright singlet metal-to-ligand charge transfer (^1^MLCT) state to the triplet long-lived ^3^MLCT state occurred through a cascade of processes within 300 fs after photoexcitation. Solvent response, internal vibrational relaxation, internal conversion, and intersystem crossing were hypothesized to be the primary events orchestrating singlet-to-triplet conversion in [Ru­(bpy)_3_]^2+^. Although this study could not further disentangle contributions of each process to the formation of the thermalized excited state, it strongly demonstrated the wealth of early time dynamics during the photocatalyst activation and pointed to its role in the outcomes of the photoredox reactions, including final products distribution and reduced quantum yields.

**Figure 7:**
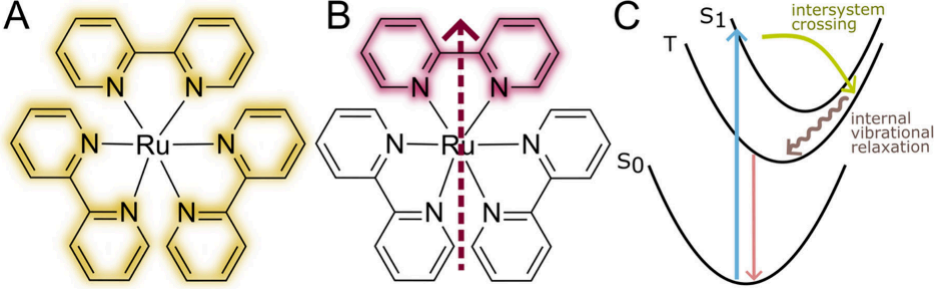
Excitation redistribution in [Ru­(bpy)_3_]^2+^. (A), (B) Anisotropy transient absorption can detect changes in transition dipole moment orientation. Localization of excitation on a single bpy moiety reduces symmetry of the complex from D_3_ to C_2_ creating a distinct transition dipole moment (red). (C) Ultrafast excitation relaxation processes in Ru and Ir complexes.

A follow-up study further clarified excitation redistribution in [Ru­(bpy)_3_]^2+^. Using anisotropy as an additional degree of control in the fs TA study, McCusker et al. experimentally probed charge localization in the excited photocatalyst.^134^ By separating contributions of two relative polarizations, i.e., parallel and perpendicular arrangements of pump and probe beams, to the TA signal, it was possible to detect reduction in molecular symmetry of [Ru­(bpy)_3_]^2+^ after photoexcitation, indicating evolution of charge from being evenly distributed among the ligands to localization on a single bpy moiety. Moreover, it was found that solvation is concurrent with charge redistribution. At the same time, once the anisotropy contribution to TA was removed, solvent dependence disappeared, suggesting that pure population dynamics, i.e., transition of the photocatalyst through a manifold of the excited states, is dictated by the intrinsic catalyst’s properties.

Later ultrafast studies provided detailed understanding of the interplay between nonthermally equilibrated excited states of precious metal complexes. It was established that initial spin-allowed optical excitation of singlet excited state of the metal is followed by the fast (∼70 fs) intersystem crossing to the optically forbidden triplet excited state and subsequent internal vibrational relaxation within this state (100 fs).^135^ The capability of transient absorption to probe triplet states through the excited state dynamics allows for the disentangling of complex pathways following the excitation of precious metal complexes.

Today, a growing body of ultrafast studies on individual photoredox reactions lays the foundation for the generalization of the observed trends across various chemical reactions.^9^ Pump–probe studies encompassing multiple reaction regimes, such as variable light conditions, sets of catalysts, or solvents, provide especially valuable insights into core mechanisms of photoredox processes. Building upon previous observations of situational reactivity of radical anions, a recent study employed optical and near-IR transient absorption spectroscopy to address these ambiguities (Figure 8).^136^ While their short excited-state lifetimes are insufficient for effective contact with the substrate, these catalytically active species are known to modify remarkably strong chemical bonds. Such reactivity could be partially attributed to the formation of the longer-lived photoactive product in oxygen-rich environments. However, this product could not facilitate higher-potential photoredox processes, suggesting that alternate mechanisms were at play in radical anion photochemistry. This comprehensive ultrafast study of different catalytic regimes delineated radical anion photoredox pathways and provided a universal framework for catalyst reactivity.

**Figure 8:**
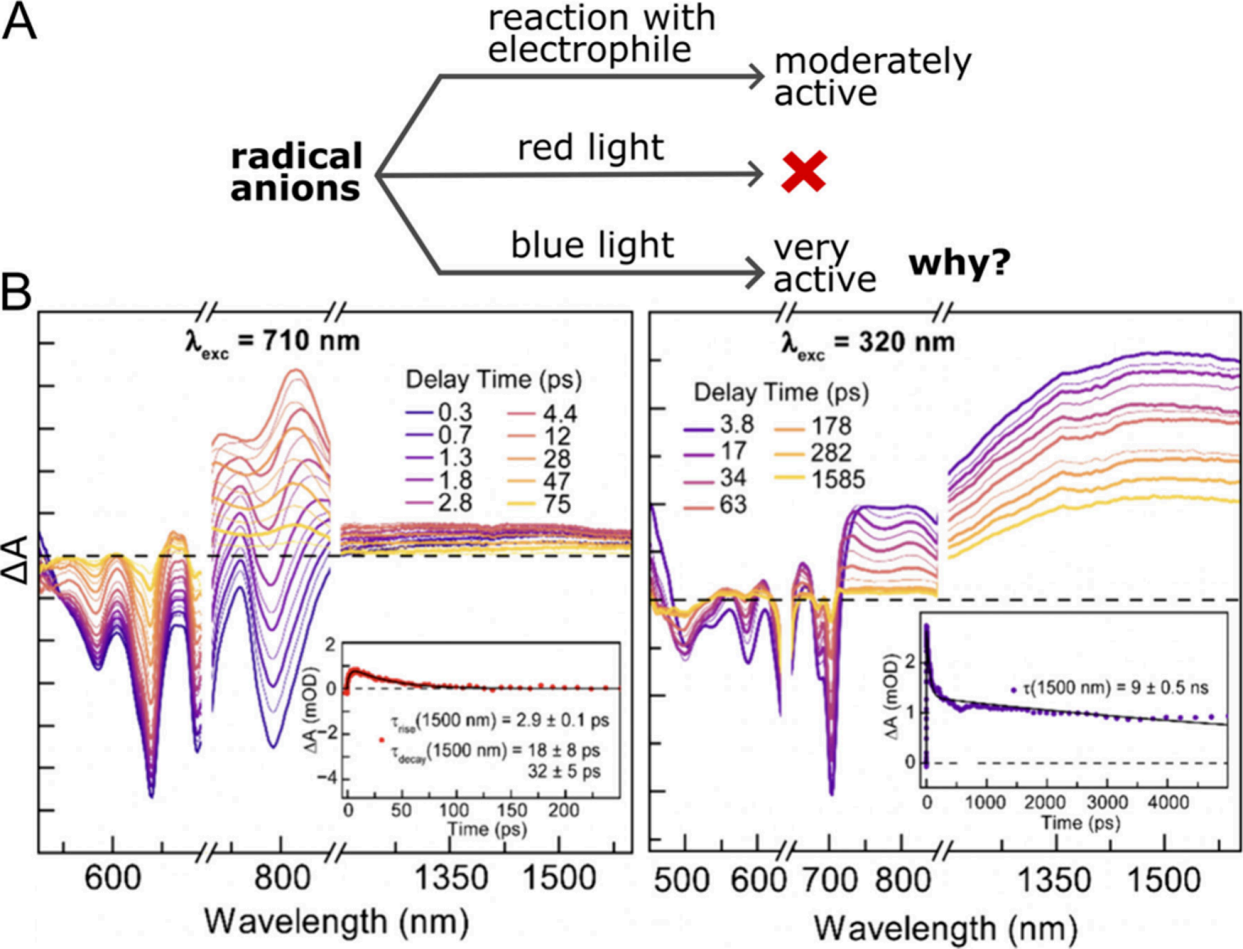
A unified framework of radical anion photoredox reactivity. (A) Summary of radical anion chemical properties. (B) Transient absorption spectra of radical anion with pump at 710 nm (left) and 320 nm (right). Inset: decay kinetics at 1500 nm. Adapted with permission from ref 136. Copyright 2025 American Chemical Society.

Different reactivity modes were identified through comparison of two excitation regimes, namely, low energy (red) and high energy (blue) pumps. While visible regions of TA spectra exhibited little variation between the two conditions, near-IR dynamics were drastically different upon blue excitation, a novel ESA long-lived feature appeared, suggesting formation of a new potentially photoactive product. Interestingly, ultrafast examination of other common radical anions revealed similar near-IR features along with the dynamics inherent to each particular compound. Further comparison of the near-IR signal to that of known photoactive catalysts led to the conclusion that a solvated electron is formed as a product of intricate interactions between radical anions and the solvent. Excited state near-IR dynamics of solvated electrons perfectly matched the one observed during blue excitation of anion radicals. This meticulous ultrafast examination spanning a range of excitation wavelengths and chemical species yielded a comprehensive understanding of radical anion photochemistry. In short, the study demonstrated that radical anions themselves do not directly facilitate photoredox transformations. Instead, their apparent reactivity is due to other photocatalytically active species formed as a result of interactions with oxygen or solvents.

Thus, far, ultrafast pump–probe investigations across the nanostructure, material, and purely chemical domains have clearly shown the viability of leveraging intrinsic atomic-scale mechanisms for existing and novel downstream applications. As core properties of quantum dots, organic photovoltaic devices, and photoredox catalysts become optimized for particular real-world implementations, even more elusive phenomena that can transparently elucidate the quantum nature of chemical systems are attracting increased attention in the ultrafast community.^137,138^ Such effects, including entanglement and coherence, are rooted in many-body, electron–electron, and electron–phonon interactions.^139^ Often ancillary to the primary process, these behaviors might be visible as faint signals in pump–probe measurements, if at all.^140^ Reliable detection of such ephemeral phenomena is a current goal of the field, requiring improved precision and novel strategies. Ultrafast spectroscopy seems to be uniquely capable of elucidating these elusive phenomena.

The deepening entrenchment of the ultrafast methods created a platform for extensive theoretical investigations across chemical domains that can provide practical advantages for their general implementation. Interdisciplinary works that aim at extracting additional information from conventional transient absorption and two-dimensional spectroscopies without increasing instrumental costs are examples of how extensive data analysis can broaden the set of experimentally observable chemical phenomena or significantly decrease data collection times and allow for investigations of unstable chemical systems.^141^ Recently, P. Malý et al. devised a feasible approach to improve data quality and obtain multiparticle dynamics by addressing laser power limitations of the pump–probe spectroscopy.^142^ Conducting pump–probe measurements in a “linear power regime” has been a well-established practice for the method since high-power illumination produces multiparticle interaction signals that mix with normally desired single-particle data and are tricky to extract unambiguously. This work suggested collecting pump–probe data at several excitation densities and using it for the perturbative expansion of the high-order signal. Such a generalized approach not only allows one to disentangle multiparticle interactions but also improves quality of the single-particle data.^143^ Another frontier study led by A. Montoya-Castillo proposed to use spectral generalized master equation framework to predict evolution of two-dimensional spectra from early time data.^144^ By devising an alternate approach to apply the generalized master equation formalism to spectral data directly, it became feasible to construct 3500 ps-long dynamics from experimentally collected 150 ps data. In addition to faster acquisition times, a noise reducing procedure allowed for the removal of statistical artifacts at longer times. Both works provide feasible routes to side-step experimental constraints of nonlinear spectroscopies. Combined with rapidly developing artificial intelligence and machine learning algorithms for data analysis, such approaches might resolve a long-standing issue of identifying artifacts in spectroscopic measurements in a reliable way.^145^

Reliable observation of now elusive quantum phenomena in chemical systems requires a reshaping of our perspective. In the late 2000s, observation of coherence in natural photosynthetic complexes using 2D spectroscopy provided an indication that nonlinear light–matter interactions can potentially probe quantum effects in disordered yet organized structures.^146^ However, this exciting result still lacks definitive interpretation: although it is clear that electronic coherence is short-lived and cannot meaningfully enhance excitation energy transfer on its own, vibrational effects, be it the vibronic mixing or vibrational and vibronic coherence, are challenging to disentangle.^147,148^ A further complication is the concern that light–matter interaction itself prepares a coherent state of the system that might not be there otherwise. Resolving this outstanding challenge seems to lie in revisiting fundamental theoretical models for nonlinear spectroscopies rather than increasing the complexity of the experimental setups. Work in this direction suggested that the pump–probe method might be better suited for the present task.^149^ Additional simplification might be gained from parameter reduction by designing the measurement to specifically address the presence of quantum effects by developing a suitable witness.^150,151^ In this framework, an experimental observable is designed to test an inequality and thereby indicate whether a truly quantum effect is present. In addition, quantum effects are expected to emerge alongside delocalization in molecular aggregates.^152,153^ Extensive collaborative and interdisciplinary efforts are required to integrate theoretical models for quantum witnesses with experimental ultrafast measurements. At the implementation level, optical control of molecular spins is another rapidly evolving direction with potential for quantum sensing and quantum logic gate applications.^137,154,155^ In the past 50 years, we have observed the dramatic development of ultrafast spectroscopy from merely a time-resolved technique to a mechanistic probe. Now is the time for another leap to bridge the gap between theoretical quantum-advantage protocols and experimentally feasible measurements.
